# The PD-1/PD-L1 Pathway Affects the Expansion and Function of Cytotoxic CD8^+^ T Cells During an Acute Retroviral Infection

**DOI:** 10.3389/fimmu.2019.00054

**Published:** 2019-02-05

**Authors:** Paul David, Dominik A. Megger, Tamara Kaiser, Tanja Werner, Jia Liu, Lieping Chen, Barbara Sitek, Ulf Dittmer, Gennadiy Zelinskyy

**Affiliations:** ^1^Institute for Virology, University Hospital Essen, University of Duisburg-Essen, Essen, Germany; ^2^Medizinisches Proteom-Center, Ruhr-Universität Bochum, Bochum, Germany; ^3^Department of Infectious Diseases, Union Hospital of Tonji Medical College, Huazhong University of Science and Technology, Wuhan, China; ^4^Department of Immunobiology, Yale School of Medicine, Yale University, New Haven, CT, United States

**Keywords:** CD8 T cells, PD-1, PD-L1, retrovirus, caspase 3, apoptosis, immunoregulation

## Abstract

Cytotoxic CD8^+^ T lymphocytes (CTL) efficiently control acute virus infections but can become exhausted when a chronic infection develops. The checkpoint receptor PD-1 suppresses the functionality of virus-specific CD8^+^ T cells during chronic infection. However, the role of the PD-L1/PD-1 pathway during the acute phase of infections has not been well characterized. In the current study the effects of PD-1 or PD-L1 deficiency on the CD8^+^ T cell response against Friend retroviral (FV) infection of knockout mice was analyzed during acute infection. We observed an enhanced proliferation, functional maturation, and reduced apoptosis of effector CD8^+^ T cells in the absence of PD-1 or PD-L1. The knockout of PD-L1 had a stronger effect on the functionality of CD8^+^ T cells than that of PD-1. Augmented CTL responses were associated with an improved control of FV replication. The strong phenotype of FV-infected PD-L1 knockout mice was independent of the interaction with CD80 as an additional receptor for PD-L1. Furthermore, we performed a detailed analysis of the production of different granzymes in virus-specific CD8^+^ T cells and observed that especially the simultaneous production of multiple granzymes in individual T cells (multifunctionality) was under the control of the PD-1/PD-L1 pathway. The findings from this study allow for a better understanding of the development of antiviral cytotoxic immunity during acute viral infections.

## Introduction

Cytotoxic CD8^+^ T Lymphocytes (CTL) are crucial for controlling viruses and tumors. However, in several chronic viral infections, such as Human Immunodeficiency virus (HIV) and Hepatitis C virus (HCV) infection of humans or Lymphocytic Choriomeningitis virus (LCMV) and Friend virus (FV) infection of mice, virus-specific CD8^+^ T cells become functionally exhausted. There is compelling evidence that this T cell exhaustion contributes significantly to the establishment of viral chronicity. The functional impairment of CTLs is associated with the expression of inhibitory checkpoint receptors and is a result of signaling from these receptors after binding to their ligands (1). The therapeutic prevention of the interaction between inhibitory ligands and checkpoint receptors with blocking antibodies reconstituted the functionality of exhausted cells during chronic infection and in some malignancies (1, 2). The so far most important and best characterized inhibitory receptor is the Programmed Cell Death Receptor-1 (PD-1) and its ligand PD-L1. Interestingly, activated virus-specific CD8^+^ T cells enhance the expression of PD-1 already very early after antigen stimulation (3). During many acute viral infections activated PD-1 positive effector cells are not exhausted (4). The expansion of PD-1 expressing CD8^+^ T cells with full effector functions has been reported during the acute infections of humans with Epstein Barr virus (EBV) (5), Hepatitis C virus (HCV) (6), or Hepatitis B virus (HBV) (7) as well as in monkeys infected with Simian Immunodeficiency virus (SIV) (8), or SIV-HIV hybrid virus (SHIV) (9). In experiments, LCMV-specific CD8^+^ T cells with PD-1 knockout were transferred into LCMV-infected mice and subsequently developed exhaustion, enhanced proliferation during early infection, and enhanced apoptosis (10). Furthermore, the SIV study provides evidence that T cell receptor stimulation itself induces PD-1 expression on CD8^+^ T cells (8).

In the current study, the murine Friend retrovirus model was used to characterize the effects of PD-1 inhibitory checkpoint receptor expressed on CTLs or its ligand PD-L1 for the regulation of these cells during an acute infection. FV is an oncogenic retroviral complex that can induce erythroleukemia in susceptible mice. However, resistant mouse strains, like the C57BL/6 mice that were used in this study, mount a potent anti-viral immune response during the acute phase of infection that prevents the onset of leukemia (11). Despite this efficient initial viral immunity, FV eventually escapes from T cell mediated immune control and establishes a chronic infection (12). We previously showed that activated CD8^+^ T cells up-regulate the expression of PD-1 but remain fully functional during the first 2 weeks of FV infection (4). These cells were highly cytotoxic but sensitive to PD-1 mediated suppression. The signal that progressively induced T cell exhaustion in the CD8^+^ T cell population came from PD-L1 expressing virus-infected cells. FV infection up-regulated PD-L1 in a subset of CTL target cells, which subsequently escaped elimination by PD-1^high^ CTLs and consequently suppressed their functionality (13). In previous studies it was also shown that regulatory CD4^+^ T cells (Tregs) (14) and myeloid derived suppressor cells (MDSCs) (15) were involved in the functional down-regulation of CTLs during the late phase of acute infection. Despite growing numbers of studies analyzing the role of PD-1 during chronic infections, the functional effects of this receptor and its ligand during the early phase of antiviral immune responses remain incompletely understood. In the current study PD-1^−/−^ and PD-L1^−/−^ mice with gene defects in the respective genes were used for the analysis of the effects of the PD-1/PD-L1 pathway on the virus-specific CTL response during an acute viral infection. We observed that mainly PD-1 regulated the magnitude of the virus-specific CD8^+^ T cell response, and in this way, determined the efficacy of antiviral immunity. The current study provides new data about the functional role of inhibitory checkpoint receptors during an acute viral infection.

## Materials and Methods

### Ethics Statement

Animal experiments were performed in strict accordance with the German regulations of the Society for Laboratory Animal Science (GV-SOLAS) and the European Health Law of the Federation of Laboratory Animal Science Associations (FELASA). The protocol was approved by the North Rhine-Westphalia State Agency for Nature, Environment, and Consumer Protection (LANUV). All efforts were made to minimize suffering.

### Mice

Inbred C57BL/6 (B6) mice were maintained under pathogen free conditions. Experiments were performed using C57BL/6 (B6) mice. The relevant FV resistance genotype of B6 mice is H-2^b/b^, Fv1^b/b^, Fv2^r/r^, Rfv3^r/r^. The B6 mice were obtained from Charles River Laboratories. B6-background PD-1 deficient (PD-1^−/−^) mice were generated in Dr. Honjo's lab (16). B6-background PD-L1^−/−^ (B7-H1-KO) mice were originally generated by L.C. (17). B6.SJL-Ptprca Pep3b/BoyJ (CD45.1) B6-background mice were obtained from Charles River Laboratories. DbGagL TCR tg mice were on a C57BL/6 background and more than 90% of the CD8^+^ T cells contained a TCR specific for the DbGagL FV epitope (18, 19). DbGagL TCR tg mice were crossed with CD45.1 mice. Offspring mice were used as donors for CD8^+^ T cells in the adoptive cell transfer experiments. All mice were females at 8–16 weeks of age at the beginning of the experiments.

### Virus and Viral Infection

The FV stock used in these experiments was a FV complex containing B-tropic Friend murine leukemia helper virus (F-MuLV) and polycythemia-inducing spleen focus-forming virus free of lactate dehydrogenase-elevating virus (20, 21). The stock was prepared as a 10% spleen cell homogenate from BALB/c mice infected 14 days previously with 3,000 spleen focus-forming units of non-cloned virus stock. Experimental mice were injected intravenously with 0.3 ml of PBS containing 20,000 spleen focus-forming units of FV.

### Cell Surface and Intracellular Staining by Flow Cytometry

Cell surface staining was performed using Becton Dickinson or eBioscience reagents. Following antibodies were used: anti-CD3, anti-CD4 (RM4-5), anti-CD8 (53-6.7), anti-CD43 (1B11), anti-CD45.1 (A20), CD80 (16-10A1), anti-PD-1(J43), and IgG fluorochrome-conjugates as isotype controlls. Dead cells were detected by Fixable Viability Dyes (FVD) (ThermoFisher).

FVD positive cells were excluded from the analyses. Intracellular granzyme A (GzA-3G8.5), granzyme B (GB11, Invitrogen, Darmstadt, Germany), granzyme K (orb102688), activated caspase 3, and Ki67 stainings were performed as described (14). Data were acquired on a LSR II flow cytometer (Becton Dickinson) from 200,000 to 300,000 lymphocyte-gated events per sample. Analyses were done using FlowJo (Treestar) and FACSDiva software (Becton Dickinson). The quantity of survivor cells at day 10 was calculated by determining the portion of cells detectible at day 10 after infection from the numbers of infected cells at the peak of FV infection at day 6. These quantities were calculated for every cell population separately.

### Tetramers and Tetramer Staining

For the detection of D^b^-GagL-specific CD8^+^ T cells, spleen cells were stained with PE labeled MHC class I H2-D^b^ (Beckman Coulter, Marseille, France) tetramers specific for FV GagL peptide (18, 22) as described previously (14).

### Cell Isolation and Adoptive Cell Transfer

CD8^+^ T cells were positively isolated by magnetic activated cell sorting using 50 μl CD8a (Ly-2) microbeads (MACS, Miltenyi Biotec, Bergisch Gladbach, Germany) according to the manufacturer's instructions. Efficiency of cell separation was proven by flow cytometry (92% CD8^+^ T cells). Ten thousand purified cells were transferred into recipient WT or PD-L1^−/−^ mice by i.v. injection in 100 μl PBS.

### *In vivo* Cytotoxicity Assay

The *in vivo* CTL assay described by Barber et al. (23) was modified to measure cytotoxicity in FV-infected mice (Figure 4A). Splenocytes from naïve CD45.1 mice were loaded with 1–5 μM D^b^GagL peptide (18, 22). The peptide loaded cells were stained with 200 nM of CFSE (Molecular Probes). As a reference, splenocytes isolated from naïve CD45.1 mice were used. Splenocytes (1 × 10^7^ cells of each population) were transferred i.v. into naïve or 10 day FV-infected mice. One hour after adoptive transfer, the spleens and bone marrows from recipient mice were harvested and cell suspensions were prepared. Cell suspensions were stained with anti CD45.1 antibodies and measured by LSR II. Donor cells were distinguished from recipient cells and from one another based on CFSE positivity and on the expression of CD45.1. The percentage of killing was calculated as follows: 100 – ([(% peptide pulsed in infected/% unpulsed in infected)/(% peptide pulsed in uninfected/% unpulsed in uninfected)] × 100).

### CD80 Blockade

C57BL/6 or PD-1^−/−^ mice were infected with FV. 250 μg of anti CD80 or control rat IgG antibody (BioXCell) were administered i.p and treatment started at day 1 after infection and repeated every alternating day for a total of three injections.

### Z-VAD-FMK Treatment

C57BL/6 or PD-1^−/−^ mice were infected with FV. Z-VAD-FMK General Caspase Inhibitor (BD Pharmingen) was administered i.p used to inhibit apoptosis *in vivo*. Z-VAD-FMK (5 μg/kg) was administered i.p. at day 6 and day 7 after infection (24).

### Label-Free Quantitative Mass Spectrometry

Cells were lysed by sonication (6 10-s pulses on ice) in sample buffer (50 mM NH4HCO3; 0.1% Rapigest). Protein isolation, digestion, amino acid analysis, and liquid chromatography-tandem mass spectrometry (LC-MS/MS) analysis were performed by following previously described procedures (25). Progenesis QI for proteomics (v. 2.0.5387.52102; Non-linear Dynamics, Newcastle upon Tyne, UK) and Proteome Discoverer (v. 1.4; Thermo Scientific, Bremen, Germany) software packages were used for protein quantification and identification, respectively. For protein identification, LC-MS/MS runs were searched against the UniProt-SwissProt database (Release 2014_10; v. 2.5; 546,790 sequences) with taxonomy restriction to *mus musculus*. Further search parameters matched previously reported ones (25). Non-unique peptides associated to more than one protein accession were not used for quantification. Differentially expressed proteins (*p* < 0.05) were functionally annotated using the Database for Annotation, Visualization, and Integrated Discovery (DAVID, ver. 6.8) (26, 27).

### Statistical Analysis

Statistics comparing the two groups were done using the unpaired non-parametric *t*-test or Mann-Whitney *t*-test. When more than two groups were compared, a one-way ANOVA was used with a Tukey post-test. (GraphPad Prism software; GraphPad Software Inc., San Diego, USA). For statistical analysis of proteomics data, normalized protein abundances were exported from Progenesis QI software and transformed using arcsinh-function. Using an in-house written R script (R Foundation for Statistical Computing, Vienna, Austria), Benjamini–Hochberg corrected one-way ANOVA was used for the calculation of the FDR-corrected *p*-values as described earlier (25).

## Results

### PD-1 Regulates the Expansion of FV-Induced CD8^+^ T Cells

**Figure 1:**
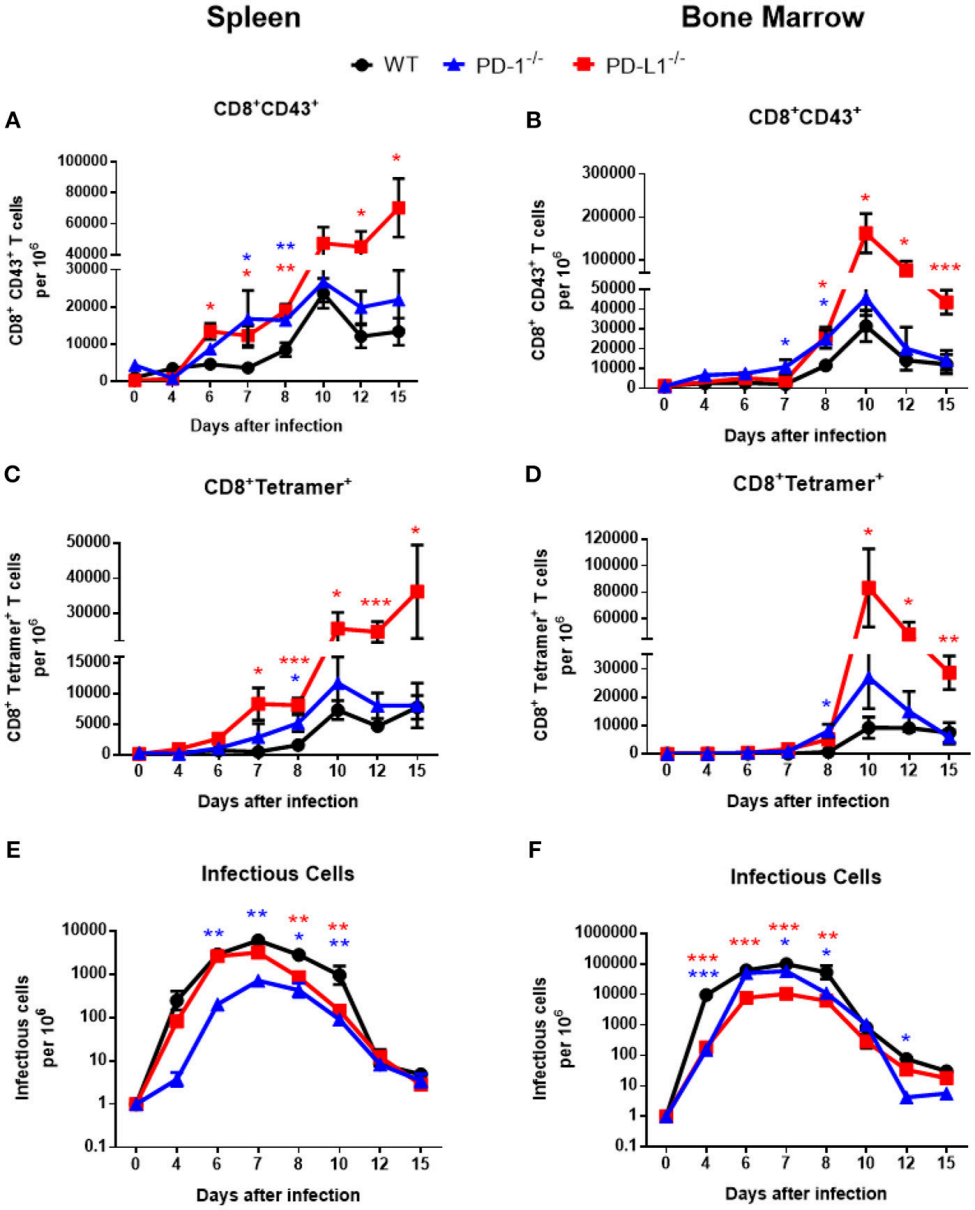
Expansion of effector CD8^+^ T cells and FV loads during acute infection of mice without PD-1 signaling. C57BL/6, PD-1^−/−^ and PD-L1^−/−^ mice were infected with FV and splenocytes and bone marrow were isolated at different time points after infection. Flow cytometry was used to detect the numbers of effector CD8^+^ T cells in spleen **(A)** and in bone marrow **(B)** expressing the activation associated glycoform of the CD43 molecules. The numbers of effector CD8^+^ specific for the FV gagL epitope were determined in spleens **(C)** and bone marrow **(D)**. Viral loads were determined in spleen **(E)** and in bone marrow **(F)**. Mean numbers plus SD of 5–9 mice are shown. Data was pooled from three independent experiments with similar results. One-way ANOVA with a Tukey post-test was used for the comparison of both KO mice strains with WT animals at every analyzed time point. Statistically significant differences between the groups (blue for comparison with PD-1^−/−^ and red for comparison with PD-L1^−/−^) are indicated (*p < 0.05, **p < 0.005, ***p < 0.0005).

Previous studies showed high expression of PD-1 on the surface of effector CD8^+^ T cells from acutely FV-infected mice (4, 28). These FV induced activated CD8^+^ T cells (CD69^+^, CD44^+^, CD62L^−^) were defined by their expression of a glycosylated form of the CD43 molecule (29). To test whether the deficiency for the PD-1/PD-L1 pathway influences acute T cell immunity, wild type (WT) C57BL/6J mice, PD-1^−/−^ and PD-L1^−/−^ mice were infected with FV. In these mice the effector CD8^+^ CD43^+^ T cells and CD8^+^ T cells specific for the H-2Db–restricted Friend murine leukemia virus–glycosylated immundominant gag epitope (tetramer^+^) (18) were characterized. Spleen and bone marrow (BM) are the organs with the highest virus replication during the acute phase of FV infection (14). Both these organs were analyzed on days 0 (uninfected), 4, 6, 7, 8, 10, 12, and 15 after FV inoculation. The expansion of effector CD8^+^CD43^+^ T cells in WT mice and both knockout (KO) mouse strains started at day 6 after infection (Figure 1A). However, in KO mice the frequencies of T cells with effector phenotypes were enhanced in the spleen at day 6 after infection. It is interesting that in the BM the population of effector CD8^+^ T cells expanded two days later at day 8 after infection (Figure 1B). At day 10 after FV infection effector CD8^+^ CD43^+^ T cell numbers peaked in both organs of all analyzed mouse strains with the exception of spleens from PD-L1^−/−^ mice. At peak expansion the frequency of effector CD8^+^ CD43^+^ T cells from PD-L1^−/−^ was 2.3 times higher than that in WT mice and in PD-1^−/−^ the frequency of effector cells was 1.4 times higher than in WT mice. In contrast to the peak levels of effector CD8^+^ T cells in WT and PD-1 KO mice at day 10, the numbers of effector cells continued to grow until day 15 after FV infection in the spleen of PD-L1 KO mice. This group was a clear exception, as the T cell contraction phase started much later than in WT or PD-1^−/−^ mice.

The kinetic of effector CD8^+^ T cells specific for the FV gagL epitope was very similar to the kinetic of the total effector CD8^+^ CD43^+^ population. The first virus-specific cells were detectable in the spleens of WT mice at day 7 after infection (Figure 1C). In both KO mouse strains the numbers of virus-specific CD8^+^ tetramer^+^ T cells were higher at this time point than in WT mice. Peak expansion of virus-specific CD8^+^ T cells was at day 10 in both organs and again frequencies were enhanced in KO mice in comparison to WT mice. In PD-L1^−/−^ mice the number of virus-specific CD8^+^ T cells was more than 3.5 times higher than in WT mice at this time point (Figure 1D). In the group with PD-1 deficiency, cell numbers of virus-specific CD8^+^ T cells were only moderately enhanced in comparison to WT mice, whereas the population of virus-specific CD8^+^ T cells was largely expanded in the group of mice with PD-L1 deficiency on day 10, 12, and 15 after infection in the spleen and BM (Figures 1C,D). Again, a T cell contraction phase was not detected in the spleen of PD-L1^−/−^ mice until 15 dpi. Thus, especially the deficiency for the PD-1 ligand resulted in a less controlled expansion of effector CD8^+^ T cells during an acute retroviral infection.

Activated effector CD8^+^ T cells eliminate FV-infected cells during acute infection. Since PD-1 and especially PD-L1 deficiency influenced the frequencies of virus-specific effector CD8^+^ T cells during acute FV infection, we analyzed if this had an effect of the viral load kinetics. The replication of FV was reduced in the spleens of PD-1^−/−^ (Figure 1E) and in the BM (Figure 1F) of PD-L1^−/−^mice before CD8^+^ T cells expanded, suggesting that these effects might not be associated with effector T cell responses. However, at day 8 after infection, as well as day 10 in the spleen, viral loads were significantly higher in WT mice compared to animals deficient for PD-1 or PD-L1, which might reflect differences in CD8^+^ T cell responses. At later time points no differences in viral loads were observed between the mouse strains, with the exception of a low viral load in PD-1 KO mice at day 12 in the BM (Figure 1F). Thus, the reduction in viral loads in both KO mice at day 8 and 10 after FV infection may be a result of an accelerated expansion of effector CD8^+^ T cells in mice without PD-1 signaling.

**Figure 2:**
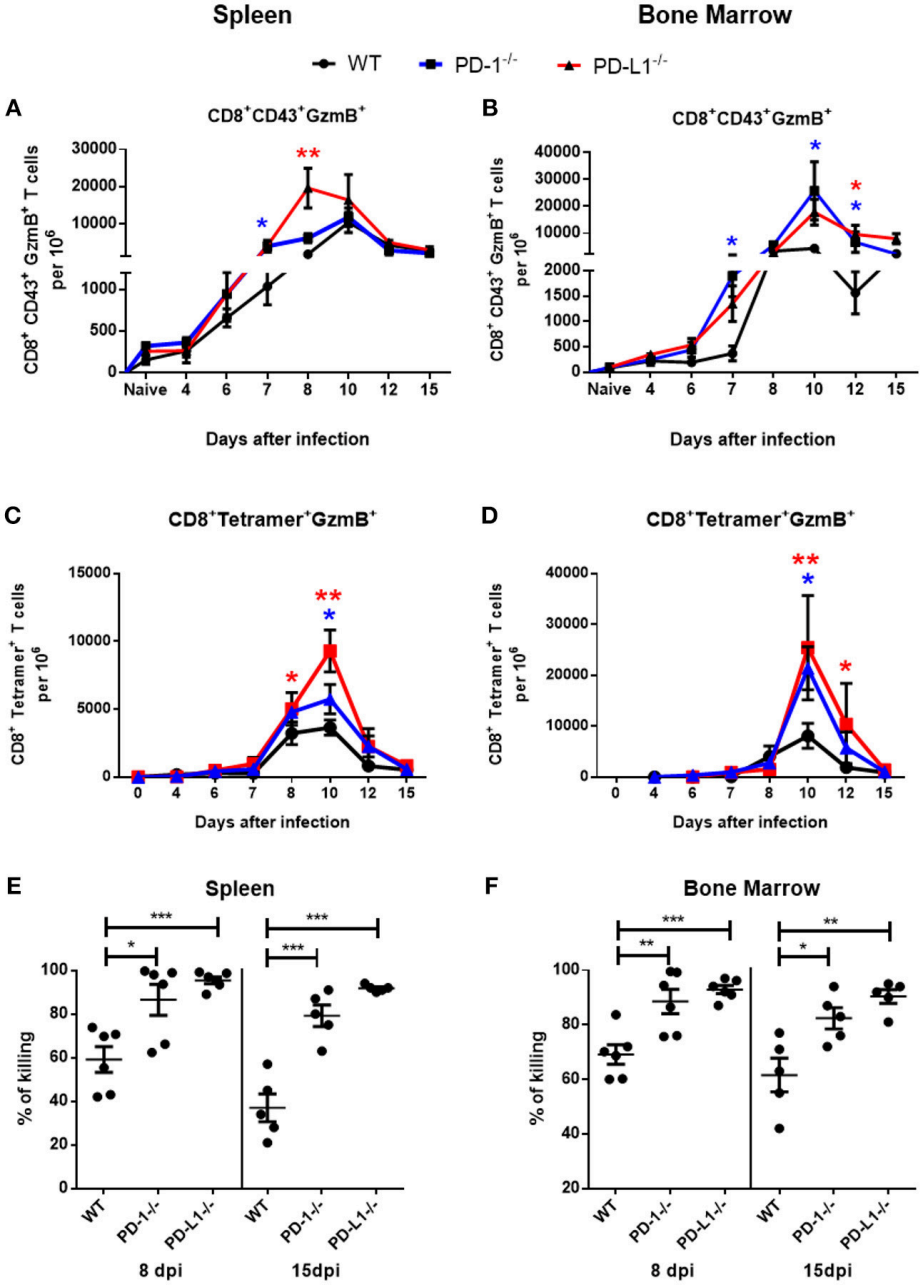
Cytotoxic CD8^+^ T cell responses in mice without PD-1 signaling. C57BL/6, PD-1^−/−^ and PD-L1^−/−^ mice were infected with FV and splenocytes and bone marrow were isolated at different time points after infection. Flow cytometry was used to detect intracellular granzyme B in effector CD8^+^ T cells (CD43^+^) and in CD8^+^ T cells specific for the FV gagL epitope (Tetramer^+^). A kinetic analysis was performed at different time points after FV infection. Shown are the numbers of effector CD8^+^ T cells expressing B (GzmB) from spleen **(A)** and bone marrow **(B)** and the numbers of virus-specific CD8^+^ tetramer^+^ CD8^+^ T cells expressing granzyme B in spleen **(C)** and bone marrow **(D)**. Each dot represents the mean number plus SEM per one million nucleated cells for a group of 5–9 mice. Data were pooled from three independent experiments with similar results. One-way ANOVA with a Tukey post-test was used for the comparison of both KO mice strains with WT animals at every analyzed time point. Statistically significant differences between the groups (blue for comparison with PD-1^−/−^ and red for comparison with PD-L1^−/−^) are indicated (*p < 0.05, **p < 0.005). CFSE labeled spleen cells from naïve CD45.1 mice were loaded with FV peptide and were injected intravenously into naïve and 8 or 15 days infected mice. As controls similar number of CD45.1 spleen cells from naïve mice without peptide were co-injected into the same recipient mice. The spleen and bone marrow cells from recipient mice were isolated 2 h after injection and analyzed for numbers of CD45.1^+^ and CFSE fluorescence. The figure shows the percentage of eliminated FV peptide-loaded donor cells in spleen **(E)** and bone marrow **(F)**. Each dot represents an individual mouse and the mean numbers and SD are indicated. Differences were analyzed by one-way ANOVA with a Tukey post-test. Statistically significant differences between the groups are indicated (*p < 0.05, **p < 0.005, ***p < 0.0005).

Activated virus-specific effector CD8^+^ T cells use the cytotoxic degranulation pathway for the elimination of FV-infected cells (30). In the next experimental step we analyzed the numbers of effector CD8^+^ T cells and virus-specific Tetramer^+^ CD8^+^ T cells producing the cytotoxic serine protease granzyme B (GzmB) in spleen and BM of infected WT, PD-1^−/−^, and PD-L1^−/−^ mice. The frequency of GzmB^+^ effector CD8^+^ CD43^+^ T cells was enhanced at day 7 after infection in spleens (Figure 2A) and in BM (Figure 2B) of PD-1^−/−^ and PD-L1^−/−^ mice in comparison to WT mice. Between day 8 and 10 post infection the numbers of effector CD8^+^ T cells producing GzmB were significantly higher in both KO mouse strains than in WT mice. Later, at day 12 and day 15 after infection, the numbers of effector cells producing GzmB in the spleen were reduced and were similar to the numbers of GzmB producing cells in WT mice. However, in BM the numbers of CD8^+^ CD43^+^ GzmB^+^ cells remained enhanced in both KO mouse strains at most of the later time points after infection (Figure 2B).

The kinetics of GzmB production in virus-specific CD8^+^ T cells were analogous to the kinetics of GzmB production in the total effector CD8^+^ T cell population. Frequencies of Tetramer^+^ CD8^+^ T cells producing GzmB were quite similar between the groups until day 8 (Figures 2C,D). However, at day 10 the numbers of cells producing GzmB were enhanced in the spleen and in the BM of mice deficient for PD-1 and PD-L1 in comparison to WT mice. In both organs the frequencies of GzmB^+^ T cells was higher in the PD-L1^−/−^ mice than in the PD-1^−/−^ mice. Based on these data we propose that PD-1 regulated not only the expansion of effector cells during the early phase of FV infection, but also their differentiation into cytotoxic effectors producing GzmB.

The main function of virus-specific CTLs is the killing of cells expressing viral antigens. In order to analyze the cytotoxic potential of CTLs we performed an *in vivo* killing assay. In this assay the elimination of transferred donor spleen cells loaded with a viral epitope peptide and labeled with CFSE were analyzed. The cytotoxicity of CD8^+^ T cells was significantly higher at day 8 and at day 15 after infection in the spleen (Figure 2E) and bone marrow (Figure 2F) of PD-1^−/−^ and PD-L1^−/−^ mice in comparison to WT mice. Interestingly, the efficacy of killing was always higher in PD-L1 KO mice than in PD-1 KO mice, reflecting the stronger expansion and effector cell activation in the PD-L1 deficient animals after FV infection.

### The Production of Different Cytotoxic Molecules in WT and PD-L1^−/−^ Mice

**Figure 3:**
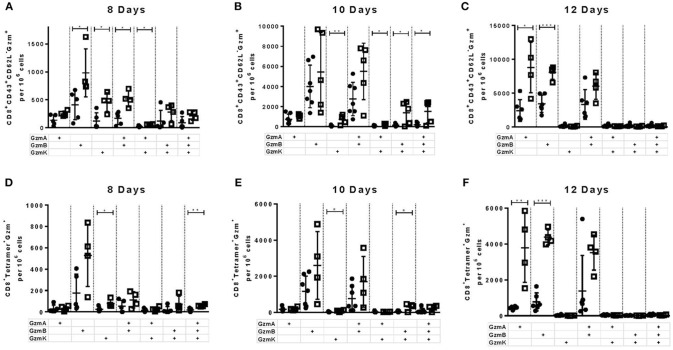
Production of different granzymes in CD8^+^ T cells from PD-L1^−/−^ mice. C57BL/6 (white columns) and PD-L1^−/−^ (black columns) mice were infected with FV and splenocytes were isolated at 8, 10, and 12 days after infection. Flow cytometry was used to detect intracellular expression of GzmA, GzmB, and GzmK in effector CD8^+^ T cells (CD43^+^) and in CD8^+^ T cells specific for the FV gagL epitope (Tetramer^+^) from the spleen. The analysis was performed at day 8, 10, and 12 after FV infection. Shown are the numbers of all effector CD43^+^ CD8^+^ T cells **(A–C)** and virus-specific Tetramer+CD8^+^ **(D–F)** expressing single, double, or all three granzymes together. Each dot represents an individual mouse and the mean numbers and SD are indicated. Data were pooled from two independent experiments with similar results. Differences were analyzed by unpaired *t*-test (*p < 0.05, **p < 0.005, ***p < 0.0005).

In previous experiments we observed that the deficiency of the PD-1 ligand had a more profound influence on the expansion and function of virus-specific CD8^+^ T cells than the deficiency of the PD-1 receptor. Therefore, in the following experiments we decided to focus on the role of PD-L1 on the functionality of cytotoxic CD8^+^ T cells. In these cells the effects of PD-L1 knockout on the production of different cytotoxic molecules was characterized in greater detail. It is known that polyfunctional CD8^+^ T cells simultaneously producing different cytokines correlates with the long-term control of HIV in elite controllers (31). However, the role of polyfunctionality regarding the production of different granzymes by T cells has not been characterized. The granzymes produced by antiviral CTLs have a different spectrum of target proteins and mediate a diverse range of cytotoxic, proinflammatory, and antiviral functions (32). To characterize the simultaneous production of different granzymes, the frequency of CD8^+^ CD43^+^ T cells expressing granzyme A (GzmA), GzmB, and granzyme K (GzmK) were determined in the spleen of WT and PD-L1^−/−^ mice at day 8 (Figure 3A), day 10 (Figure 3B), and day 12 (Figure 3C) after FV infection. Significantly more CTLs from PD-L1^−/−^ than WT mice produced GzmB or GzmK at day 8 after FV infection (Figure 3A). The frequencies of CD8^+^ CD43^+^ T cells, which were GzmA^+^ GzmB^+^ or GzmB^+^ GzmK^+^ double-positive, were also significantly higher in the PD-L1^−/−^ mouse strain. Two days later at day 10 after infection the frequencies of effector cells producing all three granzymes were also significantly higher in mice without PD-L1. At day 12 after infection significant differences were observed only in the frequencies of total effector cells producing single GzmA and GzmB. The population of FV-specific Tetramer^+^ CD8^+^ T cells was also analyzed for the production of different granzymes molecules. At day 8 after infection significantly more virus-specific CD8^+^ T cells from the spleens of PD-L1^−/−^ than WT mice produced GzmK or all three Gzms simultaneously (Figure 3D). Similar results were obtained on day 10 after infection (Figure 3E). Interesting, at the late time point day 12 after FV infection significant differences were only found for the production of single Gzms (GzmA, GzmB) (Figure 3F). The analysis of the PD-L1 effects on the production of Gzms showed a clear influence of the pathway on Gzm expression during the acute phase of antiviral T cell responses. During the early expansion of T cells and control of FV infection the PD-1 signaling pathway suppressed the simultaneous production of different Gzms in effector CD8^+^ cells.

**Figure 4:**
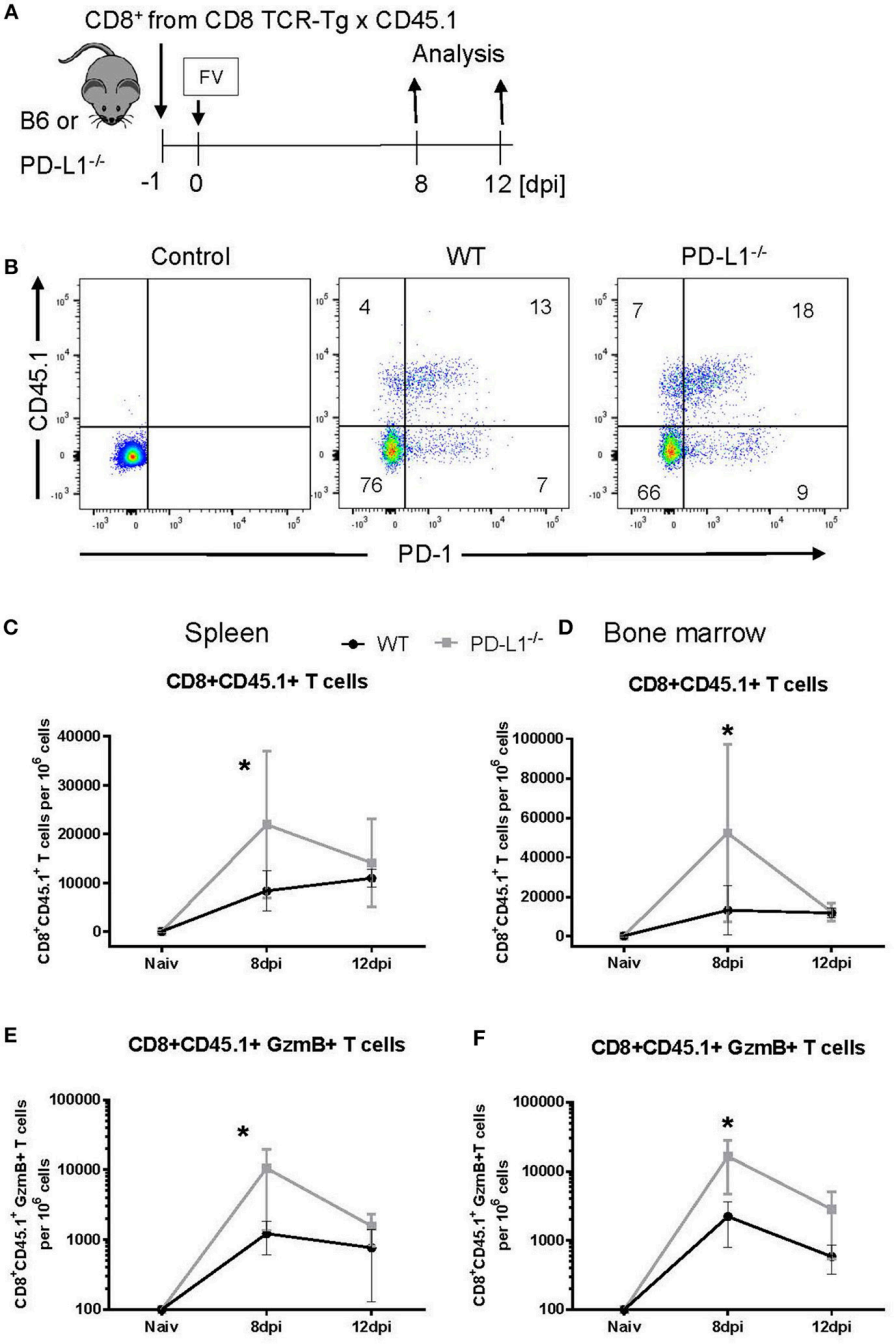
Expansion of transferred CD8^+^ T cells in PD-L1^−/−^ mice. CD8^+^ T cells were isolated from CD45.1 × TCR Tg mice and adoptively transferred into WT and PD-L1^−/−^ mice. Recipient animals were infected with FV on the next day after CD8^+^ T cell transfer **(A)**. Flow cytometry was used to detect the transferred donor CD8^+^ T cells (CD8^+^ CD45.1^+^). A representative dot plot shows the IgG isotype control for CD45.1 and PD-1 stining on CD8^+^ T cells, CD8^+^ T cells from the spleen of WT and PD-L1^−/−^ recipient mice on day 8 after FV infection **(B)**. The frequency of CD45.1^+^ CD8^+^ donor cells in the spleen **(C)** and bone marrow **(D)**, and frequency of CD45.1^+^ CD8^+^ donor cells expressing granzyme B in the spleen **(E)** and bone marrow **(F)** of 8- and 12-day infected recipient mice were determined. Mean numbers plus SD of 4–7 mice are shown. Data was pooled from two independent experiments with similar results. Unpaired *t*-test was used for the analysis of differences at every time point. Statistically significant differences between the groups are indicated (*p < 0.05).

Our current characterization of antiviral CTL responses in PD-1^−/−^ and PD-L1^−/−^ mice suggests a suppressive effect of PD-1 signaling on CD8^+^ T cell activation, expansion and contraction during the acute phase of a virus infection. In the PD-1 and PD-L1 KO mice all cells are deficient for these molecules, so we cannot distinguish between direct or indirect effects on CD8^+^ T cells. For example, the absence of PD-L1 has a significant effect on the differentiation of myeloid cells (33). The following experiment was designed to test the direct influence of PD-L1 on the expansion of effector CD8^+^ T cells. CD8^+^ T cells were isolated from the spleens of CD45.1 × TCR-Tg mice (T cell receptor transgenic mice, with CD8^+^ T cells that recognize the immunodominant gagL epitope of FV) and were transferred into WT or PD-L1^−/−^ mice 1 day prior to FV infection (Figure 4A). The expansion of transferred cells was characterized on day 8 and day 12 after infection by staining for CD45.1^+^ CD8^+^ T cells. Most of the expanded CD8^+^ CD45.1^+^ T cells from 8 days FV-infected WT and PD-L1-/- mice upregulated PD-1 on the cell surface (Figure 4B). Thus, this checkpoint receptor may be involved in the regulation of expanded virus-specific cells from donor mice. At day 8 after infection, the numbers of CD8^+^ T cells were significantly higher in the spleen and BM of infected PD-L1^−/−^ mice as compared to WT mice (Figures 4C,D). Also numbers of CD8^+^CD45.1^+^ T cells producing GzmB in the spleen and BM of PD-L1^−/−^ mice were significantly higher than in WT mice at day 8 after FV infection (Figures 4E,F). In contrast, at day 12 after infection the numbers of transferred virus-specific CD8^+^ T cells and the numbers of cells producing of GzmB were similar in both groups of mice. Thus, the transfer experiment confirmed the data from PD-L1^−/−^ mice that the expansion and functional maturation of CD8^+^ T cells during the early phase of an acute infection is regulated by the PD-L1/PD-1 pathway.

### Proteome Analysis of Transferred CD8^+^ T Cells Isolated From WT and PD-L1^−/−^ Mice

**Figure 5:**
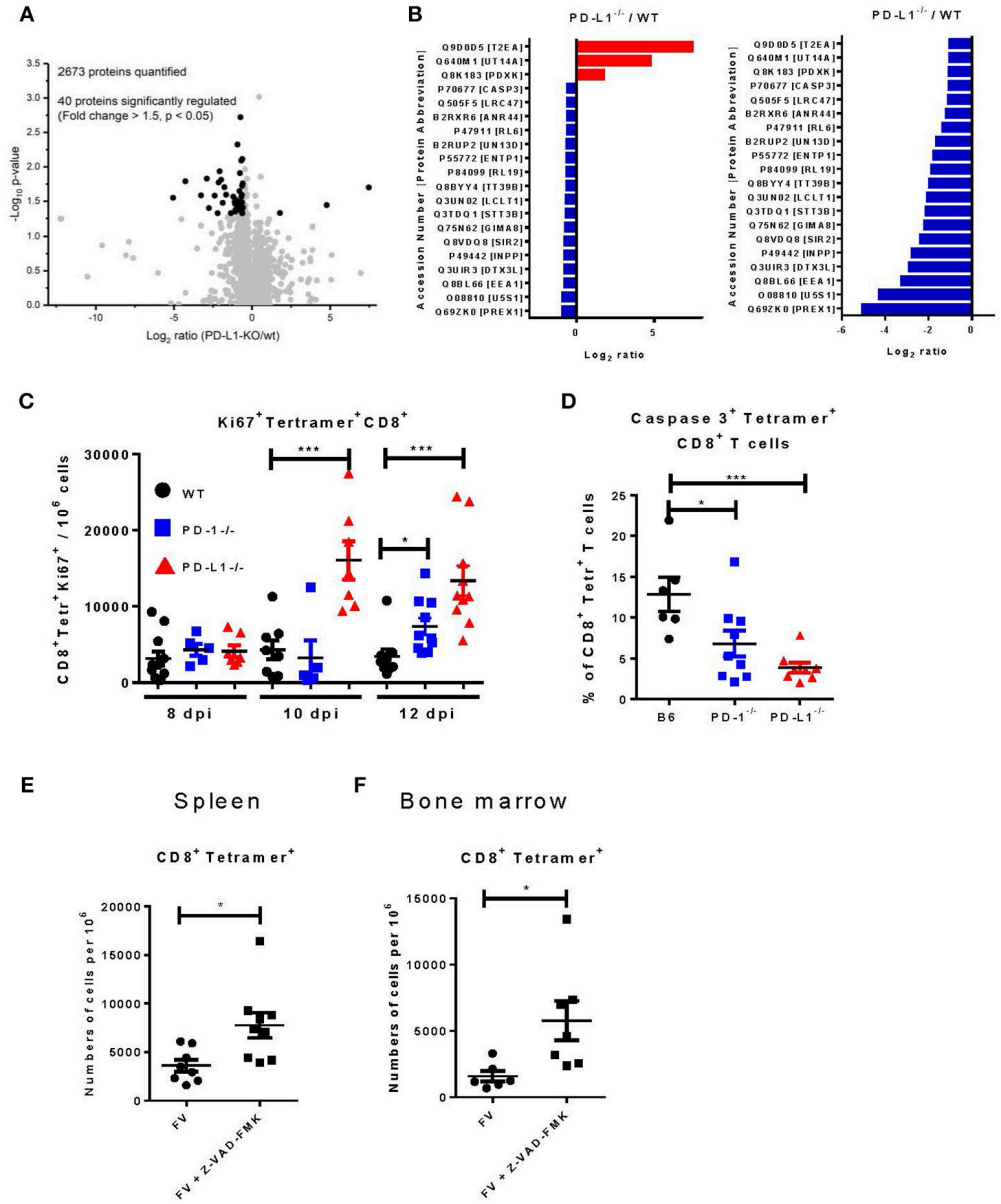
Proteome analysis, proliferation, and apoptosis of virus-specific CD8^+^ T cells in PD-L1^−/−^ mice. CD8^+^ T cells were isolated from CD45.1 × TCR Tg mice and adoptively transferred into WT or PD-L1^−/−^ mice. One day later recipient animals were infected with FV. CD8^+^ CD45.1^+^ T cells were sorted from spleens of WT and PD-L1^−/−^ mice and lysated for proteome analysis at day 12 after FV infection. **(A)** Volcano plot showing the comparison of protein expression in CD8^+^ CD45.1^+^ T cells isolated from WT and PD-L1^−/−^ mice. Significantly regulated proteins are highlighted as black dots. **(B)** The list is showing significantly regulated proteins and the ratio of protein expression in PD-L1^−/−^ to WT for every protein. **(C)** C57BL/6, PD-1^−/−^, and PD-L1^−/−^ mice were infected with FV and splenocytes were isolated at different time points after infection. Flow cytometry was used to detect the numbers of virus-specific Tetramer^+^ CD8^+^ T cells expressing the intracellular proliferation marker Ki67. **(D)** C57BL/6, PD-1^−/−^ and PD-L1^−/−^ mice were infected with FV and splenocytes were isolated at day 8 after infection. Flow cytometry was used to detect the expression of activated caspase 3 in the cytoplasm of the virus-specific Tetramer^+^ CD8^+^ T cells. C57BL/6 mice were infected with FV and at day 6 and 7 after infection they were treated with pan-caspase inhibitor Z-WAD-FMK. Flow cytometry was used to detect numbers of virus-specific Tetramer^+^ CD8^+^ T cells in spleen **(E)** and bone marrow **(F)** of 8 days infected mice. Each dot represents an individual mouse and the mean numbers and SD are indicated. Differences were analyzed by Benjamini-Hochberg corrected one-way ANOVA **(A,B)**, one-way ANOVA with a Tukey post-test **(C,D)**, or an unpaired *t*-test **(E,F)**. Statistically significant differences between the groups are indicated (*p < 0.05, ***p < 0.0005).

We used the previously described transfer model for analyzing the influence of PD-L1 on the population of virus-specific CD8^+^ T cells. For a detailed characterization of the molecular mechanisms regulated by PD-L1, a global analysis of the CD8^+^ T cell proteome was performed by label-free quantification using liquid chromatography and tandem mass spectrometry (LC-MS/MS). FV-specific CD8^+^ T cells were isolated from TCR-Tg-CD45.1 mice and transferred into WT or PD-L1^−/−^ mice. One day after transfer the recipients were infected with FV. Expanded donor CD8^+^ CD45.1^+^ T cells were sorted from splenocytes at day 12 after FV infection. Cells were lysed and subjected to proteome analysis. 2673 proteins were quantitated and compared between CD8^+^ CD45.1^+^ T cells isolated from WT vs. PD-L1 KO mice (Supplementary Table 1. Excel data). From all these proteins, only 40 molecules significantly changed expression levels in the absence of PD-L1 (Figures 5A,B; Supplementary Table 2). The concentration of 37 proteins was reduced and only three proteins [General transcription factor IIE subunit 1 (Gtf2e1), Pyridoxal kinase (Pdxk), and U3 small nucleolar RNA-associated protein 14 homolog A (Utp14a)] were enhanced in virus-specific CD8^+^ T cells isolated from PD-L1 KO mice compared to WT mice. From these differently expressed proteins 8 are involved in regulation of the cell cycle [indicated by functional analysis using DAVID (26, 27)] and the expression of caspase 3 was reduced in cells isolated from PD-L1^−/−^ mice, suggesting that proliferation and apoptosis of T cells might be affected by PD-L1.

In the next step, we therefore studied proliferation and apoptosis of CD8^+^ T cells in mice deficient for PD-L1. We analyzed the expression of Ki67 as a marker of proliferation (34) and the expression of activated caspase 3 as marker for apoptosis (35) in virus-specific CD8^+^ T cells from the spleen and BM of WT, PD-1^−/−^, and PD-L1^−/−^ mice. At day 8 after FV infection, the frequency of CD8^+^ tetramer^+^ T cells expressing Ki67 was slightly enhanced, but not significantly different in both KO mouse strains compared to WT mice (Figure 5C). However, the number of virus-specific CD8^+^ T cells expressing Ki67 was significantly enhanced in PD-L1^−/−^ mice at day 10 and 12 after infection. In PD-1^−/−^ mice significantly enhanced numbers of proliferating CD8^+^ T cells were only observed at day 12 after infection. Thus, an enhanced proliferation of virus-specific CD8^+^ T cells between day 10 and 12 after FV infection likely explains the higher number of effector T cells in both KO mouse strains. The other possible mechanism, which determines the quantity of effector cells, is the programmed death of these cells The expression of activated caspase 3, a key enzyme for the induction of apoptosis, was analyzed in the spleen of day 8 FV infected B6, PD-1^−/−^, and PD-L1^−/−^ mice (Figure 5D). 13% of the CD8^+^ tetramer^+^ T cells expressed the activated caspase 3 enzyme in WT mice, whereas only 6% of the virus-specific CD8^+^ T cells from PD-1^−/−^ and 4% from PD-L1^−/−^ mice expressed caspase 3. This significant differences in CD8^+^ T cell number expressing the activated form of caspase 3 provides evidence that PD-1 signaling may also regulate apoptosis in virus-specific CD8^+^ T cells. To prove that apoptosis regulates the number of virus-specific CD8^+^ T cells in acutely FV-infected mice, animals were treated twice with the general caspase inhibitor Z-VAD-FMK (24) on day 6 and 7 after infection. The inhibition of caspases and subsequent reduction of apoptosis led to increased numbers of virus-specific CD8^+^ T cells in the spleen (Figure 5E) and BM (Figure 5F) of treated mice at 8 days post infection. Thus, apoptosis plays an important role in regulating CD8^+^ T cell number during acute FV infection and PD-1 signaling influences levels of apoptosis.

**Figure 6:**
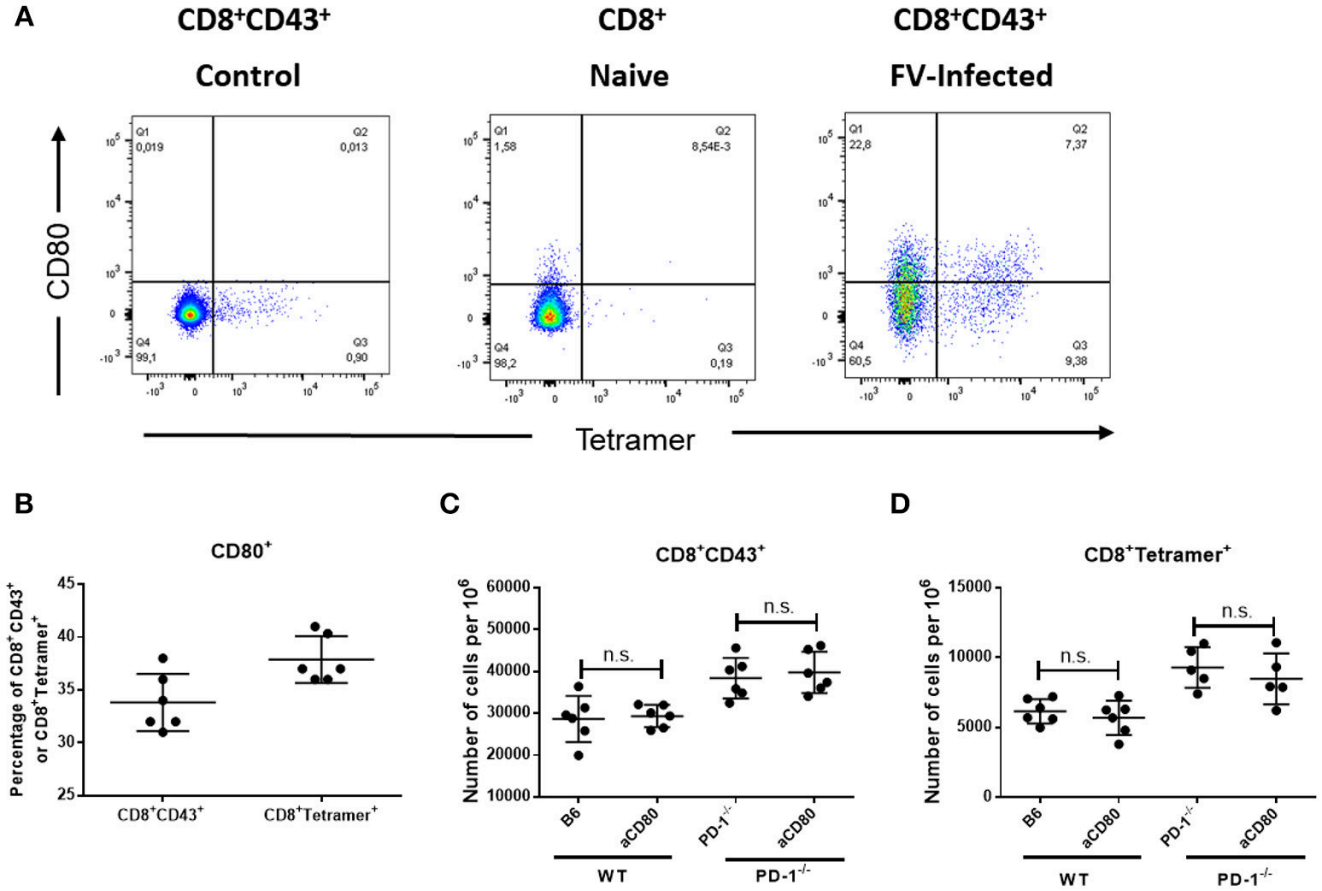
Expression of CD80 on virus-specific CD8^+^ T cells and treatment with anti-CD80 antibody. C57BL/6 mice were infected with FV and splenocytes were isolated at day 10 after infection. Flow cytometry was used for phenotypic characterization of effector CD8^+^ T cells and virus-specific Tetramer^+^ CD8^+^ T cells. **(A)**. A representative dot plot of CD8^+^ T cells from naïve mice (left) or effector CD8^+^ CD43^+^ T cells from infected mice (right) show the staining with Tetramers and CD80. **(B)**. Percentage of effector CD8^+^ T cells and virus-specific CD8^+^ T cells expressing CD80. **(C,D)**. C57BL/6 and PD-1^−/−^ mice mice were infected with FV and treated with anti-CD80 antibody. Flow cytometry was used to detect the frequency of effector CD8^+^ T cells and CD8^+^ T cells specific for the FV gagL epitope (Tetramer^+^) in anti-CD80 treated and non-treated animals. Each dot represents an individual mouse and the mean numbers and SD are indicated. Two independent experiments with similar results were performed. Differences were analyzed by unpaired *t*-test.

Our current data show that both PD-1 receptor deficiency as well as PD-1 ligand deficiency influences CD8^+^ T cell responses during an acute viral infection. Interestingly, the effect of PD-L1 knockout was stronger in almost all measurements than the effect of PD-1 knockout, which raised the question what is the difference between the two mouse models. It was previously shown that CD80 expressed on activated T cells may function as additional receptor of PD-L1 and delivers suppressive signals to T cells (36). In order to determine the role of CD80 as additional inhibitory receptor, we analyzed the expression of CD80 on effector and virus-specific CD8^+^ T cells after FV infection. Approximately 34% of the effector CD8^+^CD43^+^ T cells and 38% of the virus-specific CD8^+^ tetramer^+^ CD8^+^ T cells in the spleens of 10 day infected WT mice expressed CD80 on the cell surface (Figures 6A,B). So there might be a possible role of CD80 in suppressing CD8^+^ T cell responses. However, the treatment of FV-infected WT or PD-1^−/−^ mice with the blocking anti-CD80 antibody (37) did not affect the numbers of effector CD43^+^CD8^+^ T cells or virus-specific CD8^+^ tetramer^+^ T cells in the spleen or BM of FV-infected mice (Figures 6C,D). The production of GzmB by CD8^+^ T cells and FV viral loads were also not changed after blocking CD80 (data not shown). Thus, the CD80 expression on FV-induced effector CD8^+^ T cells was not involved in the PD-L1 dependent regulation of this population.

## Discussion

The current study used the PD-1^−/−^ and PD-L1^−/−^ mouse strains for a kinetic analysis of CD8^+^ T cell responses and FV load. We observed an enhanced expansion of CD8^+^ effector T cells in both KO mouse strains during the early acute phase of FV infection (Figure 1). PD-L1/PD-1 signaling regulated the proliferation, functional maturation, and apoptosis of virus-specific CD8^+^ T cells. However, CD80 was not involved in the PD-L1-mediated suppression of CD8^+^ T cell responses. In previous studies it was shown that deficiency in the PD-1/PD-L1 pathway or the blockade of the interaction between these molecules resulted in an enhanced elimination of the adenovirus (38), LP-BM5 immunodeficiency retrovirus (39), human metapneumovirus (HMPV) induced acute respiratory infections (40), rabies virus (41), LCMV (23), and FV (13). On the other hand the absence of PD-1 suppression during acute corona virus infection (42), murine hepatitis virus strain-3 (MHV-3) (43) or LCMV infection (44, 45) lead to an enhanced immunopathology and mortality of infected animals. In the case of FV infection, the main target organs of FV replication are BM and spleen. Short-term inflammation or immunopathology in these organs are usually not life-threatening for infected animals.

It is interesting that the frequency of effector CD8^+^ T cells remained high during late acute infection in PD-L1^−/−^ mice, but not in WT or PD-1^−/−^ mice. We therefore analyzed the role of another molecule as a possible receptor for PD-L1. Previously reverse signaling from CD80 after ligation to PD-L1 was described (36). Thus, blocking anti-CD80 antibodies were used to prevent the interaction between PD-L1 and CD80 expressed on CD8^+^ T cells. However, the treatment of WT and PD-1^−/−^ mice with anti-CD80 antibodies did not lead to a detectible modulation of CD8^+^ T cell responses. The regulation of the functionality of CD8^+^ T cells is one important aspect of PD-1/PD-L1 signaling. In the LCMV model it was previously noted that deficiency for PD-1 led to a reduced production of IFN-γ in virus-specific CD8^+^ T cells after stimulation with viral antigens (10). This observation was interpreted as an enhanced accumulation of “exhausted” terminally differentiated cells, which expressed the inhibitory receptors LAG3, CD160, 2B4, TIGIT, and Tim-3. In the FV model we previously showed that the population of PD-1^high^ CD8^+^ T cells, which also co-expressed other inhibitory receptors during the course of infection, produced significantly less cytokines in response to antigen re-stimulation than PD-1^low^ CD8^+^ T cells (10). In contrast, they produced high amounts of granzymes and were responsible for the elimination of FV-infected target cells. Thus, the terminal differentiation was associated with an enhanced cytotoxic activity of CD8^+^ T cells. The co-expression of different inhibitory checkpoint receptors on terminally differentiated CD8^+^ T cells is an essential mechanisms for the control of potentially pathogenic CTLs. Interestingly, PD-1 also plays an important role in the regulation of longterm memory CD8^+^ T cells (10). Currently it remains elusive why PD-L1 and PD-1 KO mice show such different CD8^+^ T cell responses and how these molecules influence the differentiation of the memory pool of CD8^+^ T cells during the later phase of viral infections.

The effect of the PD-1 receptor on the proliferation of effector CD8^+^ T cells is one of the known mechanisms regulating the numbers of CTLs in infected organ (38). Apoptosis is another mechanism that determines the quantity of activated effector cells. The phenomenon of activation induced cell death (AICD) which is involved in the pathogenesis of autoimmune disorders also determines the magnitude of Influenza virus-specific CD8^+^ T cells (46). Apoptosis plays a key role in dampening immune responses and for establishing long-lasting T cell memory during the contraction phase of antiviral immune responses (47). In previous studies it was shown that PD-L1 deficiency was associated with diminished expression of the pro-apoptotic molecule BCL-2-interacting mediator of cell death (Bim) in activated antigen-specific CD8^+^ T cells (48). Moreover, a similar reduction of Bim in PD-1^+^ CD8^+^ T cells was observed in melanoma patients successfully treated with anti-PD-1 antibodies (49). However, the role of apoptosis during the expansion phase of virus-specific CTLs is not well-characterized. Activated caspase 3 is the important executor molecule managing the apoptosis process (50). This enzyme cleaves different intracellular substrate molecules and starts the cascade of irreversible changes ultimately resulting in programmed cell death. Previous studies showed that the presence of caspase 3 is a phenotypic marker of activated effector T cells and not always associated with apoptosis of these cells (51). Our findings, based on proteome analysis and flow cytometry, suggest that the level of activated caspase 3 in effector CD8^+^ T cells was regulated by the PD-1 receptor. Effector cells that expanded without PD-1/PD-L1 regulation expressed reduced levels of activated caspase 3 in the cytoplasm. Cells with high levels of caspase 3 expression were predisposed to apoptotic death (35). The treatment of FV-infected mice with the low molecular pan-caspase inhibitor Z/VAD-FMK resulted in enhanced numbers of virus-specific CD8^+^ T cells during the early phase FV infection. Thus, apoptosis was involved in regulating numbers of expanded effector T cells. The regulation of T cell apoptosis through PD-1/PD-L1 molecules is most likely an important mechanism determining the magnitude of the CTL responses in infected organs. The quantity of effector CD8^+^ T cells is one important aspect of the PD-1 regulation, whereas the functional maturation toward cytotoxic T cells is another. In the current study, we analyzed the role of PD-1 signaling for the ability of CD8^+^ CTLs to simultaneously produce the different cytotoxic effector molecules granzyme A, B, and K. Interestingly, effector CD8^+^ T cells that produced two or three granzymes simultaneously were impaired by the PD-1 checkpoint mechanism. Deficiency for PD-L1 clearly enhanced numbers and presence of polyfunctional cytotoxic cells in FV-infected organs. Thus, PD-1 signaling not only regulates the quantity of antiviral T cells through the inhibition of proliferation and the induction of apoptosis, but is also critical for inhibiting the differentiation of these cells into polyfunctional cytotoxic T cells. These observations from KO mice allow us to better understand the role of PD-1 during the early phase of antiviral immune responses and may provide new strategies for the development of successful immunmodulatotry treatments of viral infections or tumors and for the development of efficient prophylactic and therapeutic vaccines.

## Author Contributions

PD and DM: data collection, data analysis and interpretation, drafting the article. TK and TW: data collection, data analysis and interpretation. JL: data collection, data analysis and interpretation, drafting the article. LC: supplied the animals, critical revision of the article. BS: data analysis and interpretation, critical revision of the article. UD: conception or design of the work, critical revision of the article. GZ: conception or design of the work, data analysis and interpretation, drafting the article, critical revision of the article.

### Conflict of Interest Statement

The authors declare that the research was conducted in the absence of any commercial or financial relationships that could be construed as a potential conflict of interest.
